# Utility of mono-exponential, bi-exponential, and stretched exponential signal models of intravoxel incoherent motion (IVIM) to predict prognosis and survival risk in laryngeal and hypopharyngeal squamous cell carcinoma (LHSCC) patients after chemoradiotherapy

**DOI:** 10.1007/s11604-023-01399-x

**Published:** 2023-02-27

**Authors:** Ya Zhang, Dehong Luo, Wei Guo, Zhou Liu, Xinming Zhao

**Affiliations:** 1 Department of Radiology, National Cancer Center/National Clinical Research Center for Cancer/Cancer Hospital, Chinese Academy of Medical Sciences and Peking Union Medical College, Beijing, 100021 China; 2 Department of Radiology, National Cancer Center/National Clinical Research Center for Cancer/Cancer Hospital & Shenzhen Hospital, Chinese Academy of Medical Sciences and Peking Union Medical College, Shenzhen, 518116 China; 3 Department of Radiology, Peking University Third Hospital, Beijing, 100191 China

**Keywords:** Intravoxel incoherent motion (IVIM), Laryngeal and hypopharyngeal squamous cell carcinoma (LHSCC), Prognosis, Survival risk

## Abstract

**Purpose:**

To investigate the predictive power of mono-exponential, bi-exponential, and stretched exponential signal models of intravoxel incoherent motion (IVIM) in prognosis and survival risk of laryngeal and hypopharyngeal squamous cell carcinoma (LHSCC) patients after chemoradiotherapy.

**Materials and methods:**

Forty-five patients with laryngeal or hypopharyngeal squamous cell carcinoma were retrospectively enrolled. All patients had undergone pretreatment IVIM examination, subsequently, mean apparent diffusion coefficient (ADCmean), maximum ADC (ADCmax), minimum ADC (ADCmin) and ADCrange (ADCmax − ADCmean) by mono-exponential model, true diffusion coefficient (D), pseudo diffusion coefficient (D*), perfusion fraction (*f*) by bi-exponential model, distributed diffusion coefficient (DDC), and diffusion heterogeneity index (α) by stretched exponential model were measured. Survival data were collected for 5 years.

**Results:**

Thirty-one cases were in the treatment failure group and fourteen cases were in the local control group. Significantly lower ADCmean, ADCmax, ADCmin, D, *f*, and higher D* values were observed in the treatment failure group than in the local control group (*p* < 0.05). D* had the greatest AUC of 0.802, with sensitivity and specificity of 77.4 and 85.7% when D* was 38.85 × 10^–3^ mm^2^/s. Kaplan–Meier survival analysis showed that the curves of N stage, ADCmean, ADCmax, ADCmin, D, D*, *f*, DDC, and α values were significant. Multivariate Cox regression analysis showed ADCmean and D* were independently correlated with progression-free survival (PFS) (hazard ratio [HR] = 0.125, *p* = 0.001; HR = 1.008, *p* = 0.002, respectively).

**Conclusion:**

The pretreatment parameters of mono-exponential and bi-exponential models were significantly correlated with prognosis of LHSCC, ADCmean and D* values were independent factors for survival risk prediction.

## Introduction

Laryngeal and hypopharyngeal cancers are common head and neck tumors, and squamous cell carcinoma is by far the most common type with a dominant proportion of nearly 95%. According to National Cancer Institute (NIH) statistics, oral cavity and pharynx cancer account for 2.8% of all new cancer cases and 1.8% of all cancer deaths, while laryngeal cancer accounts for 0.7 and 0.6%, respectively. Overall, the 5-year relative survival rate for cancers of the oral cavity, pharynx, and larynx is approximately 60–66% [1]. Currently, owing to the high response rate of radiotherapy and chemotherapy, these two methods are recommended as first-line treatments to preserve organ function [2]. However, not all patients can benefit from chemoradiotherapy (CRT). Patients with the same clinical staging could have completely different responses to CRT due to heterogeneity in tumor biology. A number of studies have suggested that for patients who are recommended CRT based on current clinical staging-oriented guidelines but have a poor response to CRT, surgery may be a better choice, especially for those with locally advanced cancer [3, 4]. One study showed that for T4N0-N3 tumors, total laryngectomy demonstrated an enhanced overall survival (hazard ratio [HR] = 0.80) compared to CRT [5]. Therefore, early identification of patients with unfavorable responses and poor survival may provide an opportunity to tailor individualized treatment strategies accordingly, thus further improving overall survival [6].

With superb soft tissue resolution, no radiation, and multiple morphological and functional sequences, magnetic resonance imaging (MRI) has been widely used for the detection and diagnosis, TNM staging, treatment response evaluation, and prognosis prediction of laryngeal and hypopharyngeal squamous cell carcinoma (LHSCC) [7, 8]. Intravoxel incoherent motion diffusion weighted imaging (IVIM-DWI) is an emerging functional MR technique developed to visualize and quantify microscopic motions of water including molecular diffusion and microcirculation of blood in the capillary network, which integrates the effects of both diffusion and perfusion [9, 10]. This makes it a potentially promising tool to visualize the tumor vasculature and oxygenation status in patients with head and neck squamous cell carcinoma (HNSCC), which is essential for predicting chemoradiotherapy resistance [11]. This potential that decreased perfusion levels are associated with a higher treatment failure rate has been demonstrated in several previous studies [12–14], which may be attributed to the decreased reactivity of tumors to radiation induced free radical damage [15, 16].

The IVIM exponential model assumes the collective motion of blood water molecules in the network, flowing from one randomly-oriented capillary segment to the next (at the voxel level) during the diffusion encoding time, mimicking a collective random walk (“pseudo-diffusion”) [10]. The mono-exponential model is used to calculate the apparent diffusion coefficient (ADC) values from diffusion weighted imaging, and the bi-exponential model is used to calculate the true diffusion coefficient (D), pseudo diffusion coefficient (D*), perfusion fraction (*f*) values, and stretched exponential model for the distributed diffusion coefficient (DDC) and diffusion heterogeneity index (α) values. The measurement of ADC values can be affected by multiple factors, among which tissue perfusion and extracellular water molecular movement are the most important. The bi-exponential model idealizes the irregular motion in the human body into perfusion-related fast dispersion and slow dispersion of water molecular motion. The stretched exponential model is a new method to fit the apparent diffusion attenuation characteristics that reflect a continuous distribution [17]. To date, studies have confirmed that the high ADC values measured before treatment of HNSCC indicate the possibility of micronecrosis in the tumor area, and could be used as an indicator to predict the short-term efficacy and long-term survival of concurrent chemoradiotherapy for HNSCC [18]. To our knowledge, previous studies have focused on investigating parameters derived from IVIM using one of the aforementioned models [19–21] or on the association between the parameters of one model and treatment response to chemoradiotherapy [22–24]. Some studies have shown that the IVIM bi-exponential model or combined ADC and bi-exponential model parameters are better than or at least not inferior to ADC alone in predicting the treatment response and differential diagnosis of lymph node metastasis in HNSCC [14, 21, 22]. However, few studies have investigated the value of the three IVIM models in predicting long-term outcomes of laryngeal and hypopharyngeal carcinomas. A study of uterine cervical carcinoma showed that SCC had lower ADC, D, *f*, and DDC values and higher D* value than adenocarcinoma; the poorly differentiated group of SCC had lower D and DDC values, and adenocarcinoma had lower ADC and D values than the well-moderately differentiated group [25].

Hence, the aim of this study was to compare the value of multi-parameters derived from IVIM mono-exponential, bi-exponential, and stretched exponential signal models in predicting long-term outcomes of patients with laryngeal and hypopharyngeal carcinoma after chemoradiotherapy.

## Materials and methods

### Patients

This retrospective study was approved by the local institutional review board (Cancer Hospital, Chinese Academy of Medical Sciences/National GCP Center for Anticancer Drugs, The Independent Ethics Committee, No.: NCC2017 G-045), and informed consent was obtained from all the enrolled patients. The inclusion criteria were as follows: 1) patients who had undergone baseline MRI including IVIM examination, before any anti-tumor treatment within 2 weeks; 2) laryngoscopy revealed a mass in the larynx or hypopharynx and biopsy confirmed the histology of squamous cell carcinoma; 3) clinical workup confirmed the absence of distant metastasis; 4) each patient had received standard chemoradiotherapy after MRI examination. We excluded those patients: 1) in whom IVIM images were degraded by severe swallowing artifacts; 2) whose tumor was too small (size less than 1 cm); 3) who had received previous anti-tumor treatment of any kind, or surgery after MRI examination. All patients received concurrent chemoradiotherapy comprising intensity modulated radiotherapy (IMRT) + concurrent chemotherapy + tumor radiosensitizer sodium glycididazole (CMNa). Radical radiation therapy was performed with 6 MVX linear accelerators, 2 Gy per day, five times per week, for a total of 33 irradiations. Concurrent chemotherapy drugs were paclitaxel + liposome 270 mg (IV drip day 1) and cisplatin 40 mg (IV drip days, 2–4), starting from the first day of radiotherapy. Each cycle lasted for 21 days. Based on the inclusion and exclusion criteria, 50 patients were enrolled in this study between December 2014 and March 2016.

### Primary endpoint

All enrolled patients were followed up after the completion of standard treatment every 3 months in the first 2 years and every 6 months in the third to fifth years by at least one of the following examination methods: MRI, CT, ultrasound examination, laryngoscopy, and biopsy at our hospital or other medical institutions. Among them, 34 patients were followed up at our hospital to obtain definitive imaging examination results and prognosis conditions, while 11 patients that could not visit the hospital in-person were followed up over the telephone to obtain information regarding the development of any new complications or adverse events. The primary endpoints were treatment failure and local control over 5 years. “Treatment failure” was defined as tumor progression, tumor recurrence, distant metastasis identified using follow-up imaging methods, and tumor-related death information obtained from telephone inquiries. In contrast, “local control” was defined as absence of tumor relapse, metastasis, or tumor-related death. The time from treatment initiation at our hospital to the endpoint of the study was regarded as progression-free survival (PFS).

### MR Imaging protocol

All patients underwent 3.0 T MRI (GE Discovery MR 750, US) using an 8-channel phased array head and neck combined coil. The routine clinical MR protocol included conventional fast spin echo T1-weighted imaging with fat suppression (repetition time/echo time, TR/TE = 660/9.3 ms) before and after administration of gadodiamide contrast agent (Gd-DTPA-BMA, 0.2 ml/kg, GE Healthcare, Ireland), and fast spin echo T2-weighted imaging with fat suppression (TR/TE = 5760/88.3 ms), field of view (FOV) = 260 mm × 260 mm, slice thickness of 4 mm, and slice spacing of 0.4 mm.

IVIM sequence scanning was performed using echo planar imaging (EPI) sequence with 12 b values (0, 10, 20, 30, 50, 70, 100, 150, 200, 400, 800, and 1000 s/mm^2^). The number of collections (NEX) was set to 2 when the b value was between 0 and 200 s/mm^2^ and 3 when the b value was between 400 and 1000 s/mm^2^ (TR/TE = 250/79 ms). The remaining parameters were as follows: bandwidth = 250 kHz, FOV = 260 mm × 260 mm, matrix = 160 × 160, slice thickness = 5 mm, and slice spacing of 1 mm.

To reduce artifacts, patients received oral codeine 20 min prior to examination. To enhance the stability of the signal-to-noise ratio (SNR), the signal from each voxel within the region of interest (ROI) was integrated to obtain the total signal intensity distribution. The mean signal intensity for each b value was derived. On the GE AW 4.6 post-processing workstation, the Functool software was used to draw the ROI on DWI images with b value of 800 s/mm^2^ by Ya Zhang (a radiologist with 5 years’ experience specializing in head and neck imaging) referring to the axial T1-weighted contrast enhanced images, which were then reviewed by a senior radiologist (Dehong Luo with more than 30 years’ experience), with any disagreement resolved through discussion. A fixed circular ROI with an area of 25–50 mm^2^ was placed on the slice of the largest area of solid tumor tissue. In reference to T1-weighted, T2-weighted and T1-weighted contrast-enhanced imaging, cystic changes, necrotic areas, and hemorrhage were avoided. Measurements for each lesion were repeated for three times to obtain the average value. The model calculation was derived from D Le Bihan’s study [9]. Mono-exponential model was selected to generate ADC mean, maximum, minimum and range (maximum- minimum) values (ADCmean, ADCmax, ADCmin, ADCrange), while the bi-exponential model was selected to generate D, D*, and *f* values. In addition, stretched exponential model was used to generate DDC and α values.

1 $$\frac{S}{{S}_{0}}=\mathrm{exp }\,(-{b}^{*}ADC)$$

2 $$\frac{{S}_{b}}{{S}_{0}}=\left(1-f\right)\,\mathrm{exp}\,\left(-bD\right)+f\,exp\,\left[-b\,(D*+D)\right]$$

3 $$\frac{{S}_{b}}{{S}_{0}}=exp\,\left\{-{\left(b\times DDC\right)}^{\alpha }\right\}$$

Formula (1) is a mono-exponential model, (2) is a bi-exponential model, and (3) is a stretched exponential model. Where ${S}_{b}$ and ${S}_{0}$ are the signal intensity at the b-value of 0–1000 and 0 s/mm^2^. ${S}_{b}={S}_{0}\times {exp}^{-bD}$ is used to calculate D value, due to the hypothesis that for b values > 200 s/mm^2^, D* is obviously greater than D so that the effect on signal attenuation is negligible.

### Statistical analysis

All data were statistically analyzed using SPSS 21.0 software (IBM, USA). The measured results were represented as the mean ± standard deviation. Chi-square test was used for categorical variables and rank sum test was used for grade data. Continuous data were tested by independent sample Student’s *t*-test or Mann–Whitney *U* test. Receiver operating characteristic (ROC) curve analysis was performed for all continuous variables and the area under the curve (AUC) was calculated. Kaplan–Meier survival curve univariate analysis was performed to determine the predictors of PFS. The parameters with *p* < 0.1 were selected into the multivariate Cox regression model, and progressive forward selection was used to determine independent predictors. *p* < 0.05 (double-tailed) was considered statistically significant.

## Results

### Patient population characteristics

During the 5 years of follow-up, 4 cases were withdrawn, and 1 case of cholangiocarcinoma occurred; therefore, 45 patients were finally analyzed. The clinical characteristics of the enrolled patients (median age, 58 [35–79] years) are summarized in Table 1. There were 9 cases of laryngeal carcinoma (supraglottic region, *n* = 5; glottic region, *n* = 4) and 36 cases of hypopharyngeal carcinoma (pyriform fossa, *n* = 26; posterior pharyngeal wall, *n* = 8; postcricoid area, *n* = 2). Thirty-one cases were assigned to the “treatment failure group” because of local recurrence and progression (*n* = 22) and metastasis (*n* = 9), of which 29 cases had tumor-related death. Fourteen cases were assigned in the “local control group” (Table 1). There was no significant difference in clinical data, including age, tumor location, smoking index, T stage and tumor stage between the two groups, while the N stage was significantly different (p = 0.012).

**Table 1:** Patient characteristics and IVIM parameters of treatment failure and local control groups

Characteristic		Treatment failure group (*n* = 31)	Local control group (*n* = 14)	*p*-value
Age		57.10 ± 9.92	57.43 ± 8.80	0.915
Tumor site	Hypopharynx	26 (83.90%)	10 (71.40%)	0.334
	Larynx	5 (16.10%)	4 (28.60%)	
Smoking index*		676.61 ± 484.70	583.21 ± 268.37	0.496
T stage	1	2 (6.45%)	2 (14.29%)	0.307
	2	6 (19.35%)	3 (21.43%)	
	3	7 (22.58%)	4 (28.57%)	
	4	16 (51.61%)	5 (35.71%)	
N stage	0	4 (12.90%)	6 (42.86%)	**0.012**
	1	3 (9.68%)	3 (21.43%)	
	2	19 (61.29%)	4 (28.57%)	
	3	5 (16.13%)	1 (7.14%)	
UICC stage	I	1 (3.23%)	1 (7.14%)	0.239
	III	3 (9.68%)	4 (28.57%)	
	IVA	16 (51.61%)	5 (35.71%)	
	IVB	11 (35.48%)	4 (28.57%)	
PFS		13.97 ± 12.31	60.00 ± 0.00	**0.000**
Mono-exponential model	ADCmean (10^−3^mm^2^/s)	1.00 ± 0.33	1.31 ± 0.26	**0.005**
	ADCmax (10^−3^mm^2^/s)	1.51 ± 0.54	1.91 ± 0.33	**0.016**
	ADCmin (10^−3^mm^2^/s)	0.60 ± 0.33	0.81 ± 0.25	**0.036**
	ADCrange (10^−3^mm^2^/s)	1.28 ± 0.46	1.31 ± 0.43	0.854
Bi-exponential model	D (10^−3^mm^2^/s)	0.92 ± 0.45	1.17 ± 0.32	**0.009**
	D* (10^−3^mm^2^/s)	88.86 ± 66.48	33.27 ± 52.10	**0.001**
	*f* (%)	28 ± 17	45 ± 20	**0.01**
Stretched exponential model	DDC (10^−3^mm^2^/s)	0.24 ± 3.97	0.42 ± 2.18	0.333
	α	0.59 ± 0.16	0.89 ± 0.74	0.061

^*^Smoking index = number of cigarettes smoked per day × number of years of smoking. The bold values of *p*-value indicate that the *p*-value of this parameter was less than 0.05.

*UICC* union for international cancer control, *PFS* progression-free survival; *ADC* apparent diffusion coefficient map, *D* true diffusion coefficient, D*, pseudo diffusion coefficient, *f* perfusion fraction, *DDC* distributed diffusion coefficient, *α* diffusion heterogeneity index

### Analysis of IVIM parameters

**Fig. 1:**
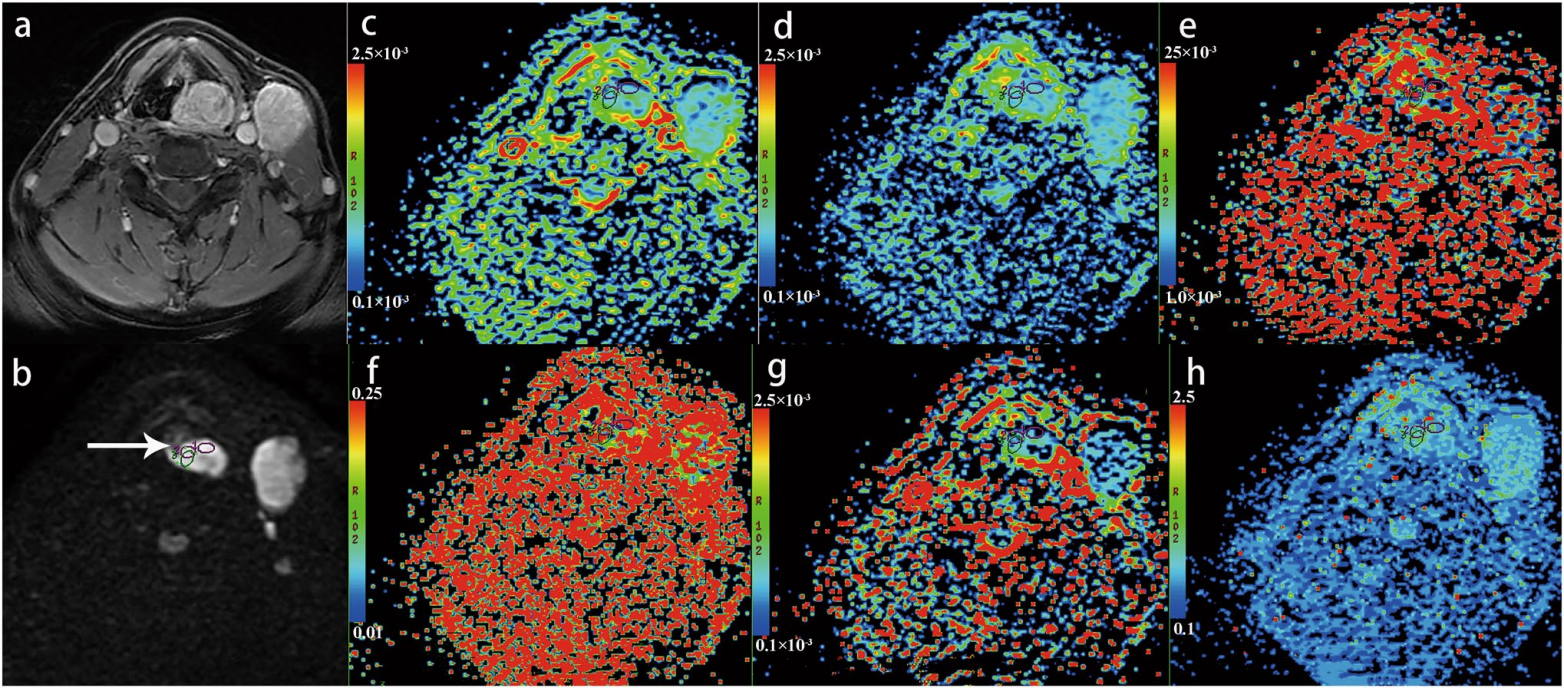
A 57-year-old man with hypopharyngeal squamous cell carcinoma is presented. Over 14 months of follow-up, the patient developed lymph node metastasis. Axial contrast-enhanced T1-weighted image (**a**) shows an obviously enhanced primary tumor at left piriform fossa and nodal mass at levels II and III of neck. **b** IVIM image (*b* = 800 s/mm^2^ level) shows the mass was hyperintense (white arrow). The three circles presented in the mass are the regions of interest (ROIs) sketched artificially in the typical area of the largest level of the mass, and are applied to each functional map. Apparent diffusion coefficient map (ADC) **c**, ADCmean = 0.58 × 10^−3^mm^2^/s, ADCmax = 0.831 × 10^−3^mm^2^/s, ADCmin = 0.12 × 10^−3^mm^2^/s, ADCrange = 1.218 × 10^−3^mm^2^/s; true diffusion coefficient (D = 0.673 × 10^−3^mm^2^/s) **d**, pseudo diffusion coefficient (D* = 67.5 × 10^−3^mm^2^/s) **e**, perfusion fraction (*f* = 26.6%) **f**, distributed diffusion coefficient (DDC = 0.513 × 10^−3^mm^2^/s) **g** and diffusion heterogeneity index (*α* = 0.729) **h** maps are shown.

**Fig. 2:**
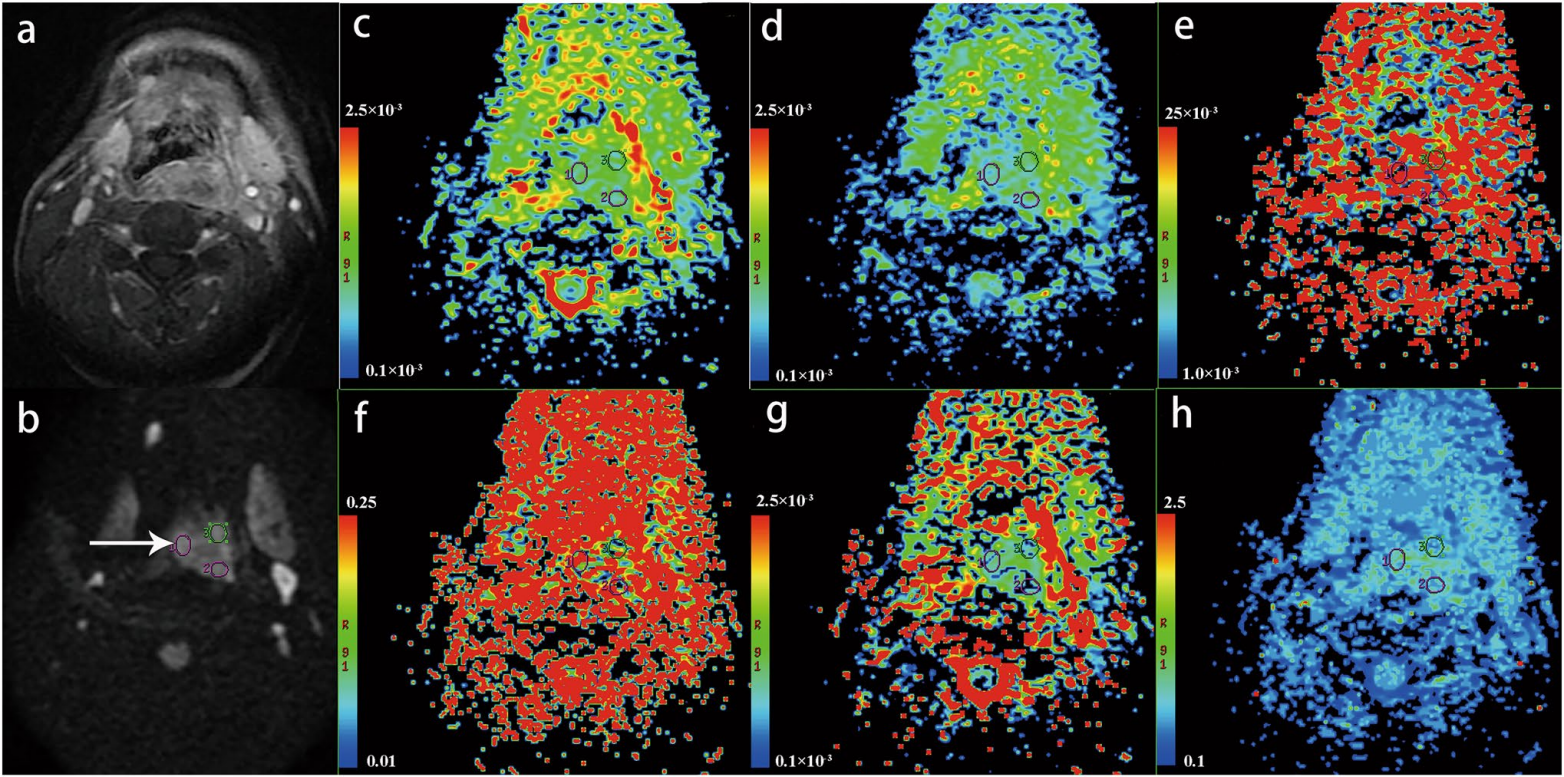
A 66-year-old man with hypopharyngeal squamous cell carcinoma is presented. Over 60 months of follow-up, no disease progression occurred. Axial contrast-enhanced T1-weighted image (**a**) shows a moderate enhanced primary tumor at posterior pharyngeal wall. IVIM image (b = 800 s/mm^2^ level) shows the mass has a slightly hyperintense signal (white arrow) (**b**). The three circles presented in the mass are the ROIs sketched artificially in the typical area of the largest level of the mass, and are applied to each functional map. ADC map (**c**), ADCmean = 0.936 × 10^−3^mm^2^/s, ADCmax = 0.1.41 × 10^−3^mm^2^/s, ADCmin = 0.695 × 10^−3^mm^2^/s, ADCrange = 0.845 × 10^−3^mm^2^/s; D = 1.01 × 10^−3^mm^2^/s (**d**), D* = 29.6 × 10^−3^mm^2^/s **(e**), *f* = 48.3% (**f**), DDC = 0.619 × 10^−3^mm^2^/s (**g**) and α = 0.751 (**h**) maps are shown.

The detailed parameters including ADCmean, ADCmax, ADCmin, ADCrange, and D, D*, *f*, DDC, and α values from the primary tumors of the two groups are summarized in Table 1. The treatment failure group showed significantly lower pretreatment ADCmean value (1.00 ± 0.33 × 10^–3^ mm^2^/s vs 1.31 ± 0.26 × 10^–3^ mm^2^/s, p = 0.005), and lower ADCmax (1.51 ± 0.54 × 10^−3^ mm^2^/s *vs* 1.91 ± 0.33 × 10^−3^ mm^2^/s, *p* = 0.016), and lower ADCmin (0.60 ± 0.33 × 10^–3^ mm^2^/s *vs* 0.81 ± 0.25 × 10^–3^ mm^2^/s, *p* = 0.036), compared to local control group; however, ADCrange did not significantly differ between the two groups. The pretreatment D value was 0.92 ± 0.45 × 10^−3^ mm^2^/s, D* value was 88.86 ± 66.48 × 10^−3^ mm^2^/s, and *f* value was 28 ± 17% in the treatment failure group. In the local control group, D value was 1.17 ± 0.32 × 10^−3^ mm^2^/s, D* value was 33.27 ± 52.10 × 10^−3^ mm^2^/s, and *f* value was 45 ± 20%. The pretreatment D and *f* values in the treatment failure group were lower than those in the local control group, and D* value was significantly higher than that in the local control group (*p* < 0.05). Two representative cases with different survival outcome were shown in Figs. 1, 2.

**Fig. 3:**
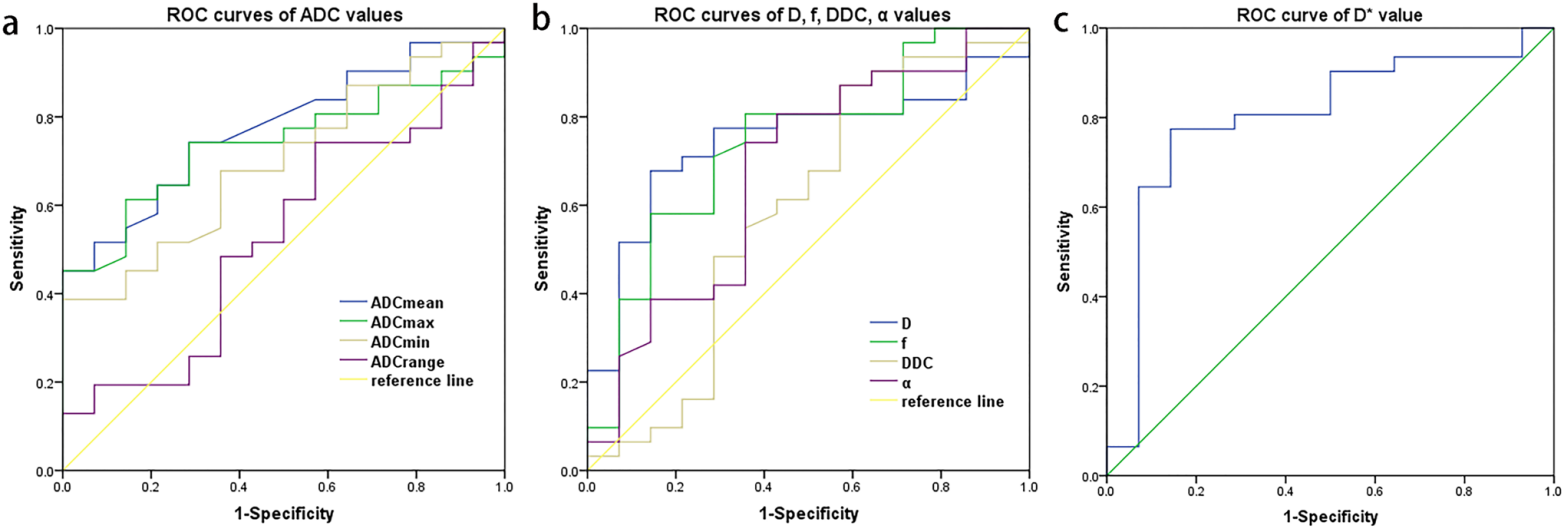
ROC curves of IVIM parameters, containing the ADC mean, max, min and range values (**a**), D,* f*, DDC, and *α* values (**b**), and D* value (**c**).

For predicting long-term prognosis, the AUC of ADCmean, ADCmax, ADCmin and ADCrange were 0.774, 0.741, 0.697 and 0.53, respectively. When the ADCmean was set as 1.15 × 10^–3^ mm^2^/s, sensitivity and specificity of 74.2 and 71.4% were obtained for predicting the local long-term prognosis. Similarly, ADCmax was set at 1.74 × 10^–3^ mm^2^/s with the sensitivity and specificity of 74.2% and 71.4%, respectively. Furthermore, ROC analysis showed that D, D*, and *f* values had significant performance with AUCs of 0.747, 0.802, and 0.741, respectively, whereas DDC and α values had fairly inferior performance with AUC of less than 0.7 (p > 0.05). D* value had the greatest diagnostic efficacy among these parameters, with the sensitivity and specificity of 77.4 and 85.7% when the threshold of D* was set at 38.85 × 10^–3^ mm^2^/s (Table 2, Fig. 3).

**Table 2:** ROC diagnostic efficiency of IVIM parameters

Parameter	AUC	*p* value	Cut-off	Sensitivity (%)	Specificity (%)
ADCmean (10^−3^mm^2^/s)	0.774	**0.004**	1.15	74.2	71.4
ADCmax (10^−3^mm^2^/s)	0.741	**0.01**	1.74	74.2	71.4
ADCmin (10^−3^mm^2^/s)	0.697	**0.036**	0.67	67.7	64.3
ADCrange (10^−3^mm^2^/s)	0.530	0.75	1.16	48.4	64.3
D (10^−3^mm^2^/s)	0.747	**0.009**	0.90	67.7	85.7
D* (10^−3^mm^2^/s)	0.802	**0.001**	38.85	77.4	85.7
*f* (%)	0.741	**0.01**	42	80.6	64.3
DDC (10^−3^mm^2^/s)	0.591	0.333	1.11	87.1	42.9
α	0.676	0.061	0.69	74.2	64.3

ROC analysis was performed for IVIM parameters to obtain the AUC, *p* value, and cut-off value, as well as the sensitivity and specificity calculated according to the cut-off value. The bold values of *p* value indicate that the *p* value of this parameter was less than 0.05

*ADC* apparent diffusion coefficient map, *D* true diffusion coefficient, D*, pseudo diffusion coefficient, *f* perfusion fraction, *DDC* distributed diffusion coefficient, *α* diffusion heterogeneity index, *ROC* receiver operating characteristic, *AUC* area under the curve

**Fig. 4:**
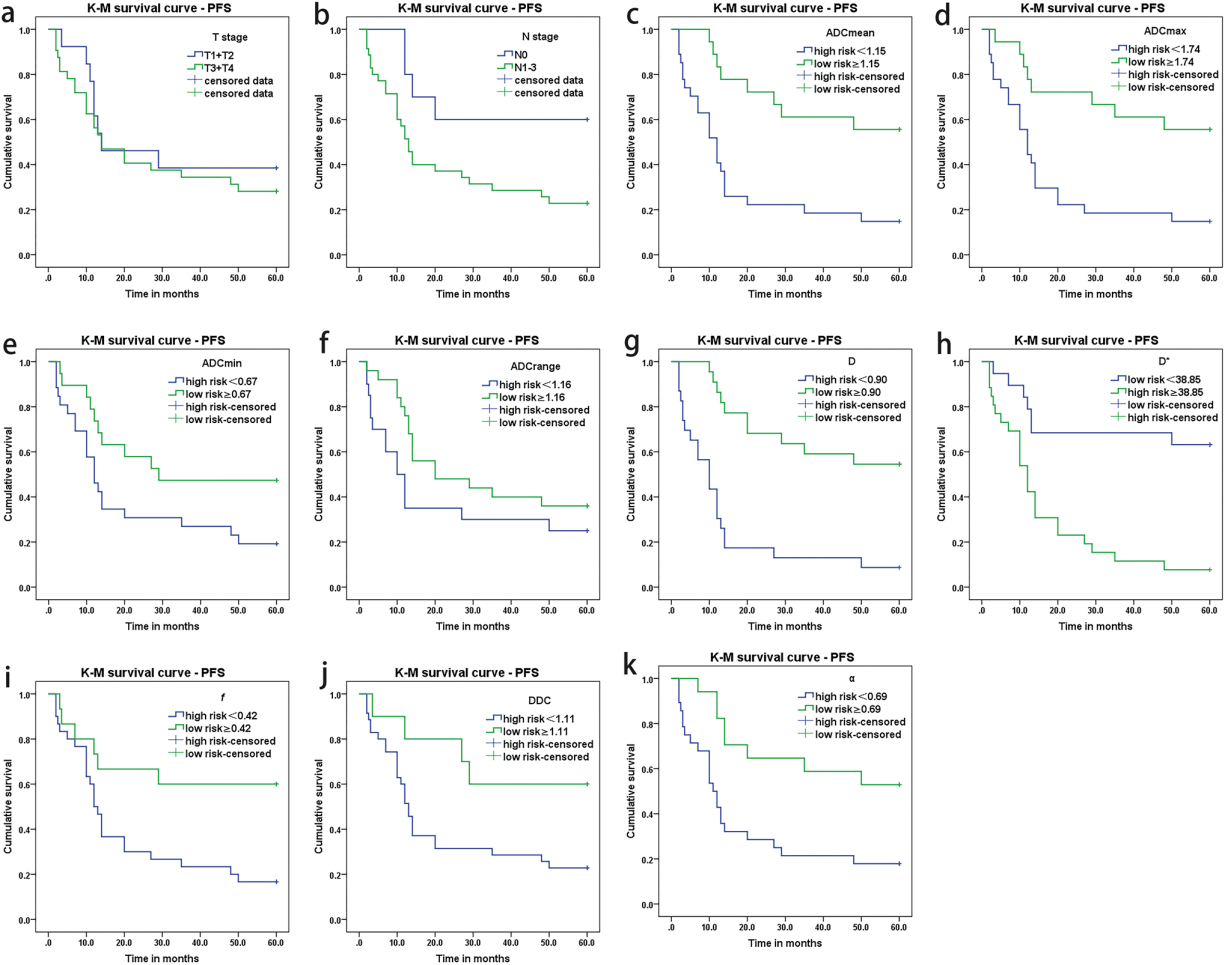
Kaplan–Meier survival analysis of clinical characteristics and IVIM parameters. **a–b** show the survivorship curve of T and N stage. **C–f** show survivorship curve of ADCmean, max, min, and range values; **g–i** show the survivorship curve of D, D*, *f* values; **j–k** show the survivorship curve of DDC and α values.

The Kaplan–Meier survival curve showed no significant difference in T stage (T1 + T2 vs T3 + T4, *p* = 0.472). However, there were significant differences in N stage (N0 vs N1-3, *p* = 0.032). The patients were divided into two groups based on the cut-off value determined by ROC analysis. Kaplan–Meier survival analysis showed that the two survivor curves based on the cut-off values of ADCmean, ADCmax, ADCmin and D, D*, *f*, DDC, and α values were found significantly different (all *p* < 0.05), except ADCrange (*p* = 0.126) (Table 3, Fig. 4). The meaningful parameters in the Kaplan–Meier survival analysis were integrated into the multivariate Cox regression analysis, and a stepwise forward analysis was adopted. Cox regression analysis showed that ADCmean (HR = 0.125, 95% CI 0.037–0.428, *p* = 0.001) and D* values (HR = 1.008, 95% CI 1.003–1.013, *p* = 0.002) were independently correlated with PFS.

**Table 3:** Kaplan–Meier survival analysis and multivariate Cox regression of clinical characteristics and IVIM parameters

Characters	Group	Total *n*	Event count *n*	Incidence rate (%)	Univariate K-M survival analysis		Multivariate Cox regression	
					Log rank (*p* value)	Median survival time/month	*p* value	HR(95%CI)
T stage	T1 + T2 T3 + T4	13 32	8 23	61.5 71.9	0.472	14 14	–	–
N stage	N0	10	4	40.0	**0.032**	– 13	0.302	–
	N1-3	35	27	77.1				
ADCmean (10^−3^mm^2^/s)	< 1.15 ≥ 1.15	27 18	23 8	85.2 44.4	**0.001**	12 –	**0.001**	0.125(0.037–0.428)
ADCmax (10^−3^mm^2^/s)	< 1.74 ≥ 1.74	27 18	23 8	85.2 44.4	**0.002**	12 –	0.51	–
ADCmin (10^−3^mm^2^/s)	< 0.67 ≥ 0.67	26 19	21 10	80.8 52.6	**0.038**	12 29	0.557	–
ADCrange (10^−3^mm^2^/s)	< 1.16 ≥ 1.16	20 25	15 16	75.0 64.0	0.126	10 20	–	–
D (10^−3^mm^2^/s)	< 0.90 ≥ 0.90	23 22	21 10	91.3 45.5	**0.000**	10 –	0.168	–
D* (10^−3^mm^2^/s)	< 38.85 ≥ 38.85	19 26	7 24	36.8 92.3	**0.000**	– 12	**0.002**	1.008(1.003–1.013)
*f* (%)	< 42 ≥ 42	30 15	25 6	83.3 40.0	**0.012**	12 –	0.806	–
DDC (10^−3^mm^2^/s)	< 1.11 ≥ 1.11	35 10	27 4	77.1 40.0	**0.035**	13 –	0.898	–
α	< 0.69 ≥ 0.69	28 17	23 8	82.1 47.1	**0.004**	11 –	0.179	–

*ADC* apparent diffusion coefficient map, *D* true diffusion coefficient, D*, pseudo diffusion coefficient, *f* perfusion fraction, *DDC* distributed diffusion coefficient, *α* diffusion heterogeneity index, *K-M* Kaplan–Meier, *CI* confidence interval, HR, hazard ratio. The bold values of *p* value indicate that the *p* value of this parameter was less than 0.05.

## Discussion

Exploration of the relationship between long-term outcomes and parameters of IVIM mono-exponential, bi-exponential, and stretched exponential signal models is of great importance to improve the interpretation of functional MRI findings and clarify the predictive value of IVIM in clinical response. In this study, we verified that pretreatment IVIM parameters were associated with long-term outcomes of patients with LHSCC treated with chemoradiotherapy.

In our study, we found that patients in the treatment failure group showed significantly lower ADCmean, ADCmax, ADCmin, D, *f* values, and higher D* values than those in the local control group. However, DDC and α values showed no significance among the two groups with different prognoses. ADCmean and D* values were significantly correlated with PFS. In addition, to our knowledge, this is the first study that showed that the predictive efficiency of IVIM parameters estimated by mono-exponential and bi-exponential models was better than that of the stretched exponential model in terms of long-term prognosis.

DWI and ADC values have gradually been applied in clinical practice. Their function in clinical diagnosis, differential diagnosis, and short-term efficacy evaluation have been confirmed by many studies [23, 26–28]. The number of bi-exponential model studies has gradually increased, and needs to be further explored for clinical application. However, only a few studies have focused on stretched exponential model in prognosis, especially in HNSCC. Currently, whether IVIM is valuable in predicting tumor prognosis and survival risk of LHSCC patients is still controversial. Hauser et al. [29] found that the ADC values of the primary lesions showed significant differences in different outcomes among patients with laryngeal and hypopharyngeal cancers, and the baseline D value of patients with better outcomes showed a lower trend. This was partially consistent with the results of this study, the D* value was the better parameter with high sensitivity and specificity in our study. In a follow-up study of patients with advanced HNSCC with positive lymph node, Simona Marzi et al. found that regional control patients had lower pretreatment D and ADC values, and higher ADC values particularly in lymph nodes than regional failure patients, which may reflect the presence of necrotic areas with lower tumor oxygenation. The baseline values of D*, *f*, and D* × *f* were lower in patients with regional control [22], which was inconsistent with our study. This could be explained by the fact that they covered the largest cross-sectional area of the lesion; however, we only covered a typical area avoiding necrotic areas. In addition, they studied lymph nodes, but we focused on primary tumors. In our study, the Functool software was segmented by *b* value = 200 s/mm^2^ to appropriately fit estimated perfusion-related parameters (D and *f*). The* f* value reflects the magnitude of the low b component, which is associated with perfusion, and is negatively correlated with the high b component, which is associated with diffusion. A study by Thomas Hauser et al. showed that the initial *f* value was significantly higher in patients with locoregional failure than locoregional control, and the initial D value did not differ significantly [23]. However, in our study, the initial *f* value was lower in the failure group than that in the local control group, which meant that the treatment failure group had lower perfusion and higher diffusion, and D value was significant in the survival analysis. This is most likely due to the fact that our failure group comprised not only local recurrence, but also metastasis and death. Furthermore, follow-up intervals were different between the two studies. In addition, since the* f* value is related to the *b *value’s setting and calculation formula, the *b* values we used (0–1000 s/mm^2^) produce Gaussian and non-Gaussian effects, which may artificially exaggerate the value and efficiency of the parameters.

The studies of Kim and King et al. [28, 30] showed that the ADC value of DWI could predict the differentiation degree of malignant tumor cells, and the differentiation degree of tumor is positively correlated with ADC value. In meningiomas, high-grade patients had lower ADC, D, *f*, and DDC values than low-grade patients; additionally, D and DDC were more efficient than *f* in differentiation [31]. These results were similar to bladder cancer [32]. Meanwhile, Zhang et al. found that α showed the highest diagnostic accuracy in differentiating benign and malignant lesions of renal cell carcinoma with AUC of 0.923, but had no impact in discriminating subtypes or grades [33]. As confirmed, tumor differentiation is correlated with treatment response and prognosis, and poorly differentiated tumors are more resistant to treatment. Hence, on the basis of IVIM parameters, we may obtain some information about the degree of tumor differentiation before biopsy. In our study, the predicted values of the mono-exponential and bi-exponential models were better than those of the stretch model. This was inconsistent with results of a previous study on cervical carcinoma [34]. The study found that DDC was the most useful parameter with an AUC of 0.948 for predicting treatment response. The reasons may be that the biological behavior of different tumor sites, tumor stage, ROI delineation method, and determination of follow-up end point events were different. In fact, there are very few comparative studies of the three models worldwide, especially regarding the long-term prognosis of tumors. Therefore, more studies are needed to confirm this hypothesis.

There were some limitations in our study. First, this was a retrospective study with a relatively small sample size. Second, the data were imbalanced, with the majority of patients having advanced-stage malignancies. Third, there were problems of recall bias and loss of follow-up, which warrants a study with larger sample size and more balanced data. Finally, the study did not deeply explore intra-tumor heterogeneity, or the correlation with the in vivo IVIM models’ functional parameters. We explored IVIM parameters with b values of 0 to 1000 s/mm^2^. A non-Gaussian effect might appear from 800 or even 600 s/mm^2^ in many tissues, including DWI data at higher b values in the IVIM analysis, which might lead to the exaggeration of *f* value, if not analyzed properly. Thus, the next step of our work is to use open-source tools such as “https://github.com/slevyrosetti/ivim-toolbox” to assess the differences in b values and model fitting [35].

## Conclusion

The quantitative parameters of the IVIM mono-exponential and bi-exponential models can predict the long-term prognosis of laryngeal and hypopharyngeal carcinomas after chemoradiotherapy, and are more efficient than the stretched exponential model. Pretreatment ADC, D, *f*, and D* values were significantly correlated with PFS, and pretreatment ADCmean and D* values were independent predictors of survival risk.

## Abbreviations

IVIM: Intravoxel incoherent motion
LHSCC: Laryngeal and hypopharyngeal squamous cell carcinoma
HNSCC: Head and neck squamous cell carcinoma
CRT: Chemoradiotherapy
ADC: Apparent diffusion coefficient
D: True diffusion coefficient
D*: Pseudo diffusion coefficient
*f*: Perfusion fraction
DDC: Distributed diffusion coefficient
α: Diffusion heterogeneity index
ROI: Region of interest
ROC: Receiver operating characteristic
AUC: Area under the curve
PFS: Progression-free survival
CI: Confidence interval
HR: Hazard ratio
